# Supplementary material for: Cost-effectiveness and cost-utility of a digital technology-driven hierarchical healthcare screening pattern in China
Source: Nat Commun. 2024 Apr 30;15:3650. doi: 10.1038/s41467-024-47211-w (PMC11061155; doi:10.1038/s41467-024-47211-w)
Supplement: Supplementary file 1 — Supplementary information [file 41467_2024_47211_MOESM1_ESM.pdf]

Cost-effectiveness and cost-utility of a digital technology-driven hierarchical healthcare screening pattern in China

Supplementary Table 1. Cost-effectiveness of different screening strategies in rural and urban settings.

Supplementary Table 2. Prevalence and transition probabilities of cataract states.

Supplementary Table 3. Variation range and distributions assumed for compliance, utilization, mortality, and other parameters.

Supplementary Table 4. Variation range and distributions assumed for the screening cost and medical cost of treating cataracts at different stages.

Supplementary Table 5. Cost computation for different screening strategies.

Supplementary Table 6. Cost computation for full examinations, treatment, and follow-up.

Supplementary Table 7. Consolidated Health Economic Evaluation Reporting Standards (CHEERS) checklist.

Supplementary Fig.1. Care pathways for organized screening and opportunistic case detection.

Supplementary Fig.2. Probabilistic sensitivity analysis of the incremental costs and incremental QALYs.

Supplementary Fig.3. Markov models for the natural progression and postoperative distributions of cataracts.

Supplementary References

Supplementary Table 1. Cost-effectiveness of different screening strategies in rural and urban settings.

	Comparison screening strategies for ICER calculation	Urban				Rural			
		Costs per person, $	Years of blindness per person	Years of blindness avoided per 100,000 people screened	ICERs, $	Costs per person, $	Years of blindness per person	Years of blindness avoided per 100,000 people screened	ICERs, $
Once-off									
Telescreening	\	2,215	0·39921	\	\	2,806	0·52060	\	\
AI screening	\	2,197	0·39605	\	\	2,750	0·50991	\	\
DH screening	Telescreening	2,189	0·39556	365	Dominating	2,737	0·50804	1,256	Dominating
	AI screening	\	\	49	Dominating	\	\	187	Dominating
Every 5 years									
Telescreening	\	2,243	0·39761	\	\	2,751	0·47131	\	\
AI screening	\	2,214	0·39413	\	\	2,637	0·45274	\	\
DH screening	Telescreening	2,193	0·39360	402	Dominating	2,610	0·44977	2,154	Dominating
	AI screening	\	\	53	Dominating	\	\	297	Dominating
Every 4 years									
Telescreening	\	2,250	0·39701	\	\	2,740	0·46076	\	\
AI screening	\	2,217	0·39346	\	\	2,613	0·44173	\	\
DH screening	Telescreening	2,192	0·39293	408	Dominating	2,583	0·43878	2,199	Dominating
	AI screening	\	\	53	Dominating	\	\	295	Dominating
Every 3 years									
Telescreening	\	2,264	0·39597	\	\	2,740	0·44544	\	\
AI screening	\	2,226	0·39237	\	\	2,592	0·42640	\	\
DH screening	Telescreening	2,194	0·39184	412	Dominating	2,557	0·42360	2,184	Dominating
	AI screening	\	\	53	Dominating	\	\	281	Dominating
Every 2 years									
Telescreening	\	2,292	0·39408	\	\	2,752	0·42354	\	\
AI screening	\	2,241	0·39051	\	\	2,567	0·40600	\	\
DH screening	Telescreening	2,196	0·39002	407	Dominating	2,520	0·40361	1,993	Dominating
	AI screening	\	\	50	Dominating	\	\	239	Dominating
Every year									
Telescreening	\	2,375	0·39017	\	\	2,866	0·39067	\	\
AI screening	\	2,288	0·38713	\	\	2,567	0·37881	\	\
DH screening	Telescreening	2,203	0·38676	341	Dominating	2,485	0·37742	1,326	Dominating
	AI screening	\	\	37	Dominating	\	\	140	Dominating

Notes: Costs are given in US dollars. Costs and years of blindness are defined as lifetime values per person, whereas years of blindness avoided and ICERs are defined as values per 100,000 people. The ICER thresholds of cost-effectiveness are $31,656 and $41,757 per QALY gained for rural and urban settings. The ICER thresholds of being highly cost-effective are $10,552 and $13,919 per QALY gained for rural and urban settings, respectively. Negative ICUR or ICER is defined as dominating. ICER=incremental cost-effectiveness ratio. DH screening=digital hierarchical screening.

Supplementary Table 2. Prevalence and transition probabilities of cataract states

	Urban settings				Rural settings			
	Prob•	Source	Range for one-way sensitivity analysis	Distributions used in the probabilistic sensitivity analysis	Prob•	Source	Range for one-way sensitivity analysis	Distributions used in the probabilistic sensitivity analysis
Senile cataracts	26•66%	(1)	Not defined	Not defined	28•79%	(1)	Not defined	Not defined
Mild	17•23%	Calculated from (2)	±10% (58•17%-71•09%)	Beta(317•78,1526•54)	19•38%	Calculated from (3)	±10% (60•57%-74•03%)	Beta(309•52,1287•95)
Moderate	6•36%	Calculated from (2)	±10% (21•47%-26•25%)	Beta(359•65,5294•21)	6•25%	Calculated from (3)	±10% (19•53%-23•87%)	Beta(360•08,5403•63)
Severe	3•07%	Calculated from (2)	±10% (10•36%-12•66%)	Beta(372•33,11761•29)	3•16%	Calculated from (3)	±10% (9•90%-12•10%)	Beta(371•95,11372•93)
Normal to mild	3•68%	Calculated from (4)	±10% (3•31%-4•05%)	Beta(369•97,9683•63)	a	a	a	a
Mild to moderate	11•25%	Calculated from (5)	±10% (10•13%-12•38%)	Beta(340•82,2688•67)	a	a	a	a
Moderate to severe	3•06%	Calculated from (5)	±10% (2•75%-3•37%)	Beta(372•36,11796•28)	a	a	a	a
POST-1 (%)	79•7%	(4)	±10% (71•73%-87•67%)	Beta(77•18,19•66)	68•60%	(3)	±10% (61•74%-75•46%)	Beta(119•94,54•90)
POST-2 (%)	13•5%	(4)	±10% (12•15%-14•85%)	Beta(332•15,2128•23)	25•32%	(3)	±10% (22•79%-27•85%)	Beta(286•63,845•39)
POST-3 (%)	6•8%	(4)	±10% (6•12%-7•48%)	Beta(357•96,4906•10)	6•08%	(3)	±10% (5•47%-6•69%)	Beta(360•73,5572•31)

Notes:
Mild cataracts: patients' best corrected visual acuity (BCVA) before surgery>0•3. Moderate cataracts: patients' BCVA before surgery was within 0•1-0•3. Severe cataracts: patients' BCVA before surgery <0•1. Referable cataracts: patients with moderate or severe cataracts. POST-1: patients' postoperative BCVA >0•3. POST-2: patients' postoperative BCVA was within 0•1-0•3. POST-3: patients' postoperative BCVA <0•1.
[a] Same as the urban setting.

Supplementary Table 3.
Variation range and distributions assumed for compliance, utilization, mortality, and other parameters.

	Urban settings				Rural settings			
	Base-case values	Source	Ranges for sensitivity analysis	Probability distribution for sensitivity analysis	Base-case values	Source	Ranges for sensitivity analysis	Probability distribution for sensitivity analysis
Compliance with telescreening...								
Community screening	90%	(6)	±10% (81%-99%)	Beta(37•51,4•17)	95%	(6)	±10% (85•50%-100%)	Beta(4•05,0•21)
Hospital referral	40%	(7)	±10% (36•00%-44•00%)	Beta(230•09,345•13)	a	a	a	a
Surgery therapy	91%	(8)	±10% (81•90%-100%)	Beta(33•66,3•33)	80%	a	±10% (72•00%-88•00%)	Beta(76•03,19•01)
Compliance with AI screening…								
AI community screening	90%	(6)	±10% (81%-99%)	Beta(37•51,4•17)	95%	(6)	±10% (85•50%-100%)	Beta(4•05,0•21)
Hospital referral	52%	(7)	±10% (46•80%-57•20%)	Beta(183•87,169•73)	a	a	a	a
Surgery therapy	91%	(8)	±10% (81•90%-100%)	Beta(33•66,3•33)	80%	a	±10% (72•00%-88•00%)	Beta(76•03,19•01)
Compliance with DH screening…								
AI smartphone screening	98%	(9)	±10% (88•20%-100•00%)	Beta(6•70,0•14)	a	a	a	a
Digital community screening	90%	(6)	±10% (81•00%-99•00%)	Beta(4•05,0•21)	95%	a	±10% (85•50%-100%)	Beta(4•05,0•21)
Hospital referral	62%	Assumed based on (7)	±10% (55•80%-68•20%)	Beta(145•36,89•09)	a	a	a	a
Surgery therapy	91%	(8)	±10% (81•90%-100%)	Beta(33•66,3•33)	80%	a	±10% (72•00%-88•00%)	Beta(76•03,19•01)
Opportunistic case finding rate	45•1%	(10)	±10% (41%-50%)	Beta(210•44,256•17)	18•90%	(6)	±10% (17•01%-20•79%)	Beta(311•35,1336•02)
Utility for…								
Normal	1•00	(11)	Not defined	Not defined	a	a	a	a
Mild	0•61	Calculated from (12)	±10% (54•90%-67•10%)	Beta(149•21,95•39)	a	a	a	a
Moderate	0•48	(12)	±10% (43•20%-52•80%)	Beta(199•28,215•88)	a	a	a	a
Severe	0•35	(12)	±10% (31•50%-38•50%)	Beta(249•34,463•07)	a	a	a	a
POST-1	0•81	Calculated from (12)	±10% (72•90%-89•10%)	Beta(72•18,16•93)	a	a	a	a
POST-2	0•55	(12)	±10% (49•50%-60•50%)	Beta(172•32,140•98)	a	a	a	a
POST-3	0•53	(12)	±10% (47•70%-58•30%)	Beta(180•02,159•64)	a	a	a	a
Discount rate	3•5%	(13)	Not defined	Not defined	a	a	a	a
DH smartphone screening								
Sensitivity	88•67%	(14)	±10% (79•80%-97•54%)	Beta(42•64,5•45)	a	a	a	a
Specificity	89•33%	(14)	±10% (80•40%-98•26%)	Beta(40•10,4•79)	a	a	a	a
AI/DH community screening								
Sensitivity	94•8%	(14)	±10% (85•32%-100%)	Beta(19•03,1•04)	a	a	a	a
Specificity	97%	(14)	±10% (87•30%-100%)	Beta(10•55,0•32)	a	a	a	a
Telescreening								
Sensitivity	95•00%	(8)	±10% (85•50%-100%)	Beta(18•26,0•96)	a	a	a	a
Specificity	97•00%	(8)	±10% (87•30%-100%)	Beta(10•55,0•33)	a	a	a	a
Mortality rate for age groups								
50-54 years	0•30%	(15)	Not defined	Not defined	a	a	a	a
55-59 years	0•45%	(15)	Not defined	Not defined	a	a	a	a
60-64 years	0•75%	(15)	Not defined	Not defined	a	a	a	a
65-69 years	1•17%	(15)	Not defined	Not defined	a	a	a	a
70-74 years	2•03%	(15)	Not defined	Not defined	a	a	a	a
75-79 years	3•56%	(15)	Not defined	Not defined	a	a	a	a
80-84 years	6•29%	(15)	Not defined	Not defined	a	a	a	a
Increased mortality risk (odds ratio)								
Cataracts	2•99	(16)	Not defined	Not defined	a	a	a	a
After cataract surgery	1•00	(17)	Not defined	Not defined	a	a	a	a

Notes:
Mild cataracts: patients' best corrected visual acuity (BCVA) before surgery>0•3. Moderate cataracts: patients' BCVA before surgery was within 0•1-0•3. Severe cataracts: patients' BCVA before surgery <0•1. Referable cataracts: patients with moderate or severe cataracts. POST-1: patients' postoperative BCVA >0•3. POST-2: patients' postoperative BCVA was within 0•1~0•3. POST-3: patients' postoperative BCVA <0•1. AI = artificial intelligence. DH screening = digital hierarchical screening.
[a] Same as the urban setting.

Supplementary Table 4
Variation range and distributions assumed for the screening cost and medical cost of treating cataracts at different stages

	Urban settings			Rural settings		
	Cost($/person)	Range for sensitivity analysis and assigned distribution	Probability distribution for sensitivity analysis	Cost($/person)	Range for sensitivity analysis and assigned distribution	Probability distribution for sensitivity analysis
Screening						
DH/AI screening	8•38	±50% ($4•19-$12•57)	Gamma(15•36,0•54)	5•88	±50% ($2•94-$8•82)	Gamma(15•36,0•38)
Telemedicine screening	15•84	±50% ($7•92-$23•76)	Gamma(15•36,1•03)	10•84	±50% ($5•42-$16•26)	Gamma(15•36,0•70)
Full ophthalmologic examination						
Normal	40•99	±20% ($32•79-$49•19)	Gamma(96•03,0•42)	155•28	±20% ($124•22-$186•34)	Gamma(96•03,1•62)
Mild	42•46	±20% ($33•97-$50•95)	Gamma(96•03,0•44)	156•75	±20% ($125•40-$188•10)	Gamma(96•03,1•63)
Moderate	344•15	±20% ($275•32-$412•98)	Gamma(96•03,3•58)	458•44	±20% ($366•75-$550•13)	Gamma(96•03,4•77)
Severe	325•67	±20% ($260•54-$390•80)	Gamma(96•03,3•39)	439•96	±20% ($351•97-$527•95)	Gamma(96•03,4•58)
Treatment and follow-up						
Moderate	1,539•61	±20% ($1,231•69-$1,847•53)	Gamma(96•03,16•03)	2,106•29	±20% ($1,685•03-$2,527•55)	Gamma(96•03,21•93)
Severe	1,615•86	±20% ($1,292•69-$1,939•03)	Gamma(96•03,16•82)	2,182•54	±20% ($1,746•03-$2,619•05)	Gamma(96•03,22•73)
Indirect costs for blindness (severe cataract and POST-3)	$3600 each year per person till death	±50% ($1,800•00-$5,400•00)	Gamma(15•36,234•28)	a	a	a

Notes:
Mild cataracts: patients' best corrected visual acuity (BCVA) before surgery>0•3. Moderate cataracts: patients' BCVA before surgery was within 0•1-0•3. Severe cataracts: patients' BCVA before surgery <0•3. POST-3: patients' postoperative BCVA <0•1. AI = artificial intelligence. DH screening = digital hierarchical screening.
[a] Same as the urban setting.

Supplementary Table 5.
Cost computation for different screening strategies.

	**Urban settings (Annualized cost, $/year)**		**Rural settings (Annualized cost, $/year)**	
	DH/AI screening ($)	Telemedicine screening ($)	DH/AI screening ($)	Telemedicine screening ($)
Direct medical costs				
AI or telemedicine platform	31,561	13,000	31,561	13,000
Equipment	2561	2561	2561	2561
Medical personnel wage	24,000	48,000	24,000	48,000
Direct nonmedical costs				
Transportation for participants and one accompanying family member	1•14	1•14	1•14	1•14
Advertisement	1224	1224	1224	1224
Indirect costs				
Income loss*	6	12	3•5	7
Number of screening people[&]	48,000	24,000	48,000	24,000
Societal costs per person	8•38	15•84	5•88	10•84

Notes:
All the costs were collected from the Finance Department and Procurement Center of the Zhongshan Ophthalmic Center. The annualized cost for fixed assets was calculated by assuming a life span of 5 years and no salvage value. AI = artificial intelligence. DH screening = digital hierarchical screening.
*Since the participants were older than 50 years old, we assumed that only their accompanying family member produced a wage loss.
[&]We assumed that in the telemedicine screening group, each patient's visit time was 5 minutes, and eye care staff worked 8 hours a day for 250 days a year; therefore approximately 24,000 patients were screened annually. In DH and AI screening, each patient was examined for 2•5 minutes, and a total of approximately 48,000 patients were screened annually.

Supplementary Table 6.
Cost computation for full examinations, treatment, and follow-up.

	**Urban settings (Annualized cost, $/year)**				**Rural settings (Annualized cost, $/year)**			
	Normal	Mild	Moderate	Severe	Normal	Mild	Moderate	Severe
Full examinations								
Direct medical costs								
Personnel wage	1•95	1•95	1•95	1•95	1•95	1•95	1•95	1•95
Examination	13•61	15•18	316•77	298•29	13•61	15•18	316•77	298•29
Direct nonmedical costs								
Transportation, food and accommodation	1•43	1•43	1•43	1•43	97•72	97•72	97•72	97•72
Indirect costs*								
Income loss	24	24	24	24	42	42	42	42
Societal costs	40•99	42•46	344•15	325•67	155•28	156•75	458•44	439•96
Treatment and follow-up								
Direct medical costs								
Personnel wage			7•8	7•8			7•8	7•8
Treatment and follow-up examinations			1,539•61	1,615•86			1,539•61	1,615•86
Direct nonmedical costs								
Transportation, food and accommodation			5•72	5•72			390•88	390•88
Indirect costs								
Income loss			96	96			168	168
Societal costs			1,649•13	1,725•38			2,106•29	2,182•54

Notes:
Mild cataracts: patients' best corrected visual acuity (BCVA) before surgery>0•3. Moderate cataracts: patients' BCVA before surgery was within 0•1~0•3. Severe cataracts: patients' BCVA before surgery <0•1.
The costs of transportation, food and accommodation for urban and rural residents and accompanying family members who visited the Zhongshan Ophthalmic Center are estimated. The examinations include visual acuity, intraocular pressure, slit lamp photography, indirect ophthalmoscopy and autorefraction for normal and mild cataract patients. Additional retinal fundus photography, optical coherence tomography and other necessary preoperative assessments are involved for moderate cataracts patients. Ultrasound biomicroscopy is added for severe cataract patients. Additionally, considering that the retina cannot be photographed clearly in severe cataracts patients, relevant examinations are performed in follow-ups after surgery.
By investigating rural and urban patients who came to the Zhongshan Ophthalmic Center, the costs of transportation, food and accommodation for rural and urban residents are estimated. Phacoemulsification and intraocular (IOL) implantations are performed on patients with referable cataracts, and they have regular follow-up examinations 1 day, 1 week, 1 month, and 6 months after surgery. We assume that the patients undergo cataract surgery for each eye within 3 months. The costs are obtained from the ZOC under the Chinese government's control and vary little from institution to institution.
*Indirect costs consist of one accompanying family member's wage loss based on the time spent and per capita daily income in rural and urban areas.

Supplementary Table 7. Consolidated Health Economic Evaluation Reporting Standards (CHEERS) checklist 2022

Topic	No.	Item	Location where item is reported
Title			
	1	Identify the study as an economic evaluation and specify the interventions being compared.	Page 1
Abstract			
	2	Provide a structured summary that highlights context, key methods, results, and alternative analyses.	Pages 3
Introduction			
Background and objectives	3	Give the context for the study, the study question, and its practical relevance for decision making in policy or practice.	Pages 4-5
Methods			
Health economic analysis plan	4	Indicate whether a health economic analysis plan was developed and where available.	Pages 8-9
Study population	5	Describe characteristics of the study population (such as age range, demographics, socioeconomic, or clinical characteristics).	Page 8, Supplementary Information
Setting and location	6	Provide relevant contextual information that may influence findings.	Pages 9
Comparators	7	Describe the interventions or strategies being compared and why chosen.	Pages 9
Perspective	8	State the perspective(s) adopted by the study and why chosen.	Page 8
Time horizon	9	State the time horizon for the study and why appropriate.	Page 8
Discount rate	10	Report the discount rate(s) and reason chosen.	Page 11
Selection of outcomes	11	Describe what outcomes were used as the measure(s) of benefit(s) and harm(s).	Page 11
Measurement of outcomes	12	Describe how outcomes used to capture benefit(s) and harm(s) were measured.	Page 11
Valuation of outcomes	13	Describe the population and methods used to measure and value outcomes.	Page 11
Measurement and valuation of resources and costs	14	Describe how costs were valued.	Page 10, Supplementary Information
Currency, price date, and conversion	15	Report the dates of the estimated resource quantities and unit costs, plus the currency and year of conversion.	Page 10
Rationale and description of model	16	If modelling is used, describe in detail and why used. Report if the model is publicly available and where it can be accessed.	Page 8
Analytics and assumptions	17	Describe any methods for analysing or statistically transforming data, any extrapolation methods, and approaches for validating any model used.	Pages 8-11, Supplementary Information
Characterising heterogeneity	18	Describe any methods used for estimating how the results of the study vary for subgroups.	Pages 8-11
Characterising distributional effects	19	Describe how impacts are distributed across different individuals or adjustments made to reflect priority populations.	Pages 8-11
Characterising uncertainty	20	Describe methods to characterise any sources of uncertainty in the analysis.	Pages 8-11, Supplementary Information
Approach to engagement with patients and others affected by the study	21	Describe any approaches to engage patients or service recipients, the general public, communities, or stakeholders (such as clinicians or payers) in the design of the study.	Pages 8-11
Results			
Study parameters	22	Report all analytic inputs (such as values, ranges, references) including uncertainty or distributional assumptions.	Pages 5-6
Summary of main results	23	Report the mean values for the main categories of costs and outcomes of interest and summarise them in the most appropriate overall measure.	Page 5
Effect of uncertainty	24	Describe how uncertainty about analytic judgments, inputs, or projections affect findings. Report the effect of choice of discount rate and time horizon, if applicable.	Page 5
Effect of engagement with patients and others affected by the study	25	Report on any difference patient/service recipient, general public, community, or stakeholder involvement made to the approach or findings of the study	Page 5
Discussion			
Study findings, limitations, generalisability, and current knowledge	26	Report key findings, limitations, ethical or equity considerations not captured, and how these could affect patients, policy, or practice.	Pages 6-8
Other relevant information			
Source of funding	27	Describe how the study was funded and any role of the funder in the identification, design, conduct, and reporting of the analysis	Page 13
Conflicts of interest	28	Report authors conflicts of interest according to journal or International Committee of Medical Journal Editors requirements.	Page 13

From: Husereau D, Drummond M, Augustovski F, et al. Consolidated Health Economic Evaluation Reporting Standards 2022 (CHEERS 2022) Explanation and Elaboration: A Report of the ISPOR CHEERS II Good Practices Task Force. Value Health 2022;25. doi:10.1016/j.jval.2021.10.008

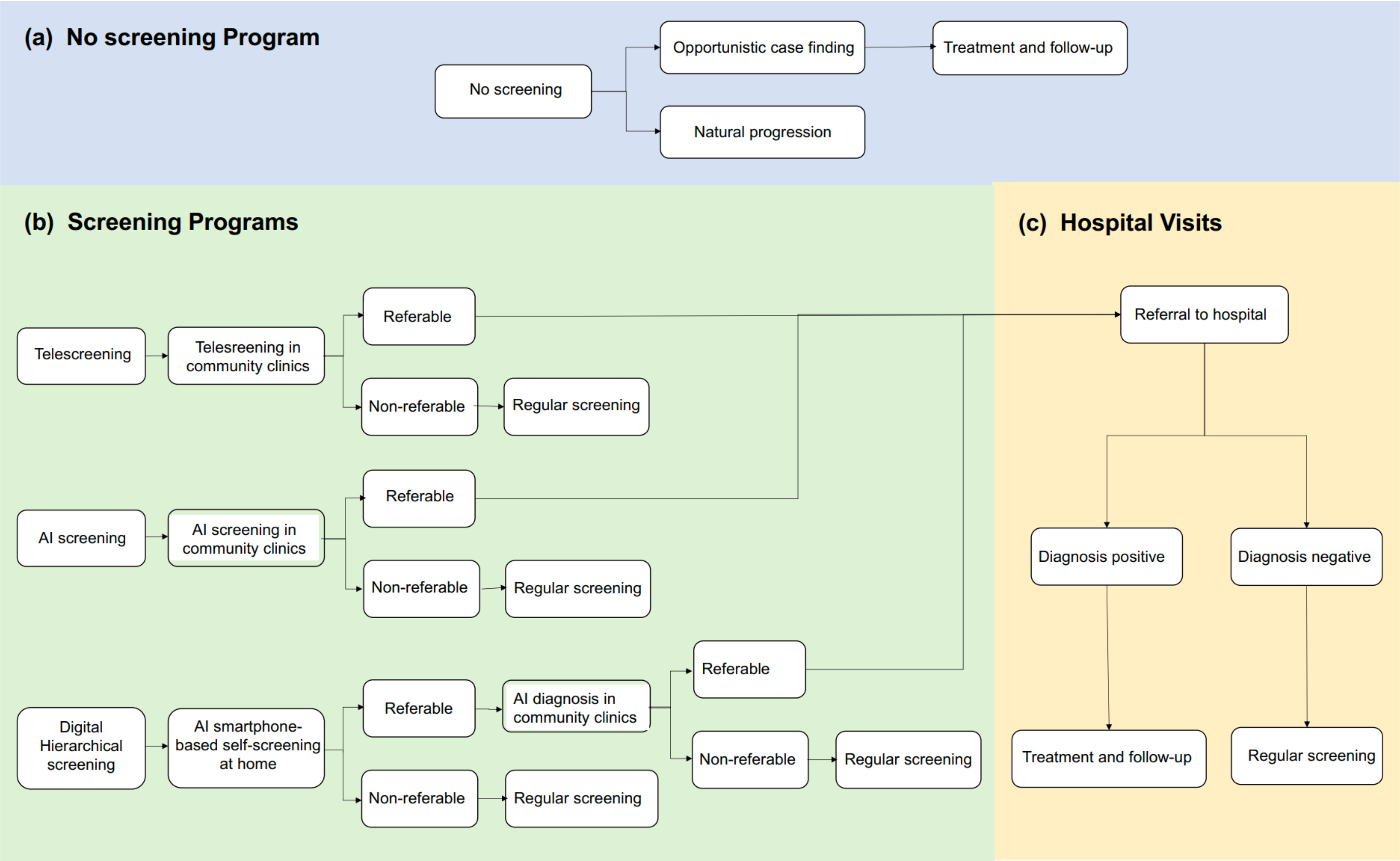

Supplementary Fig.1. Care pathways for organized screening and opportunistic case detection.
Care pathways for opportunistic case detection (a), screening programs (b) and hospital visits (c).

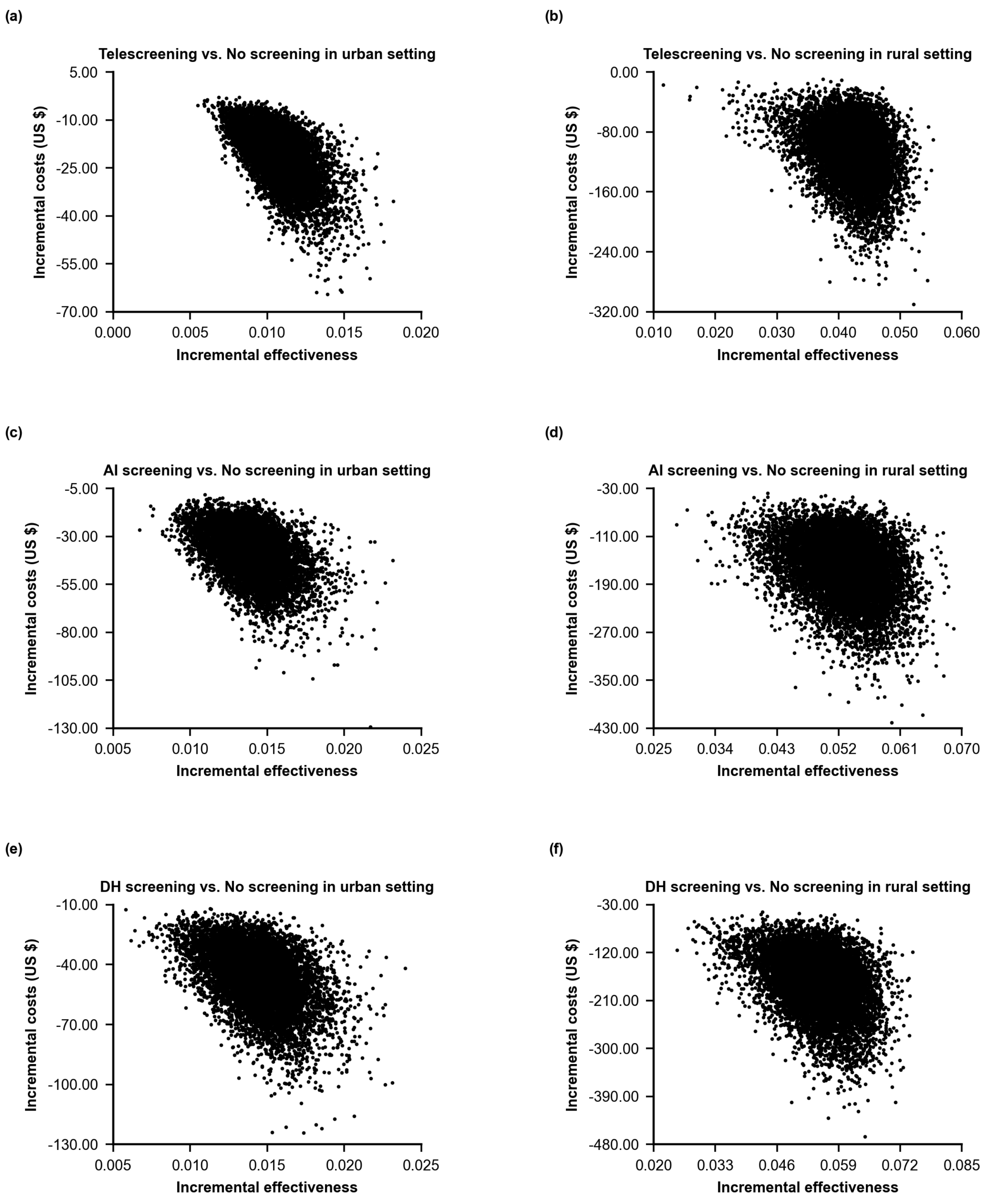

Supplementary Fig.2. Probabilistic sensitivity analysis of the incremental costs and incremental QALYs.

Costs are expressed in US dollars. Incremental benefits are defined as incremental QALYs. We performed probabilistic sensitivity analyses for telescreening vs. no screening (a and b), AI screening vs. no screening (c and d), and DH screening vs. no screening (e and f) in urban (a, c, e) and rural (b, d, f) settings, respectively. DH screening=digital hierarchical screening. QALYs= quality-adjusted life-years. GDP= gross domestic product. AI: artificial intelligence.

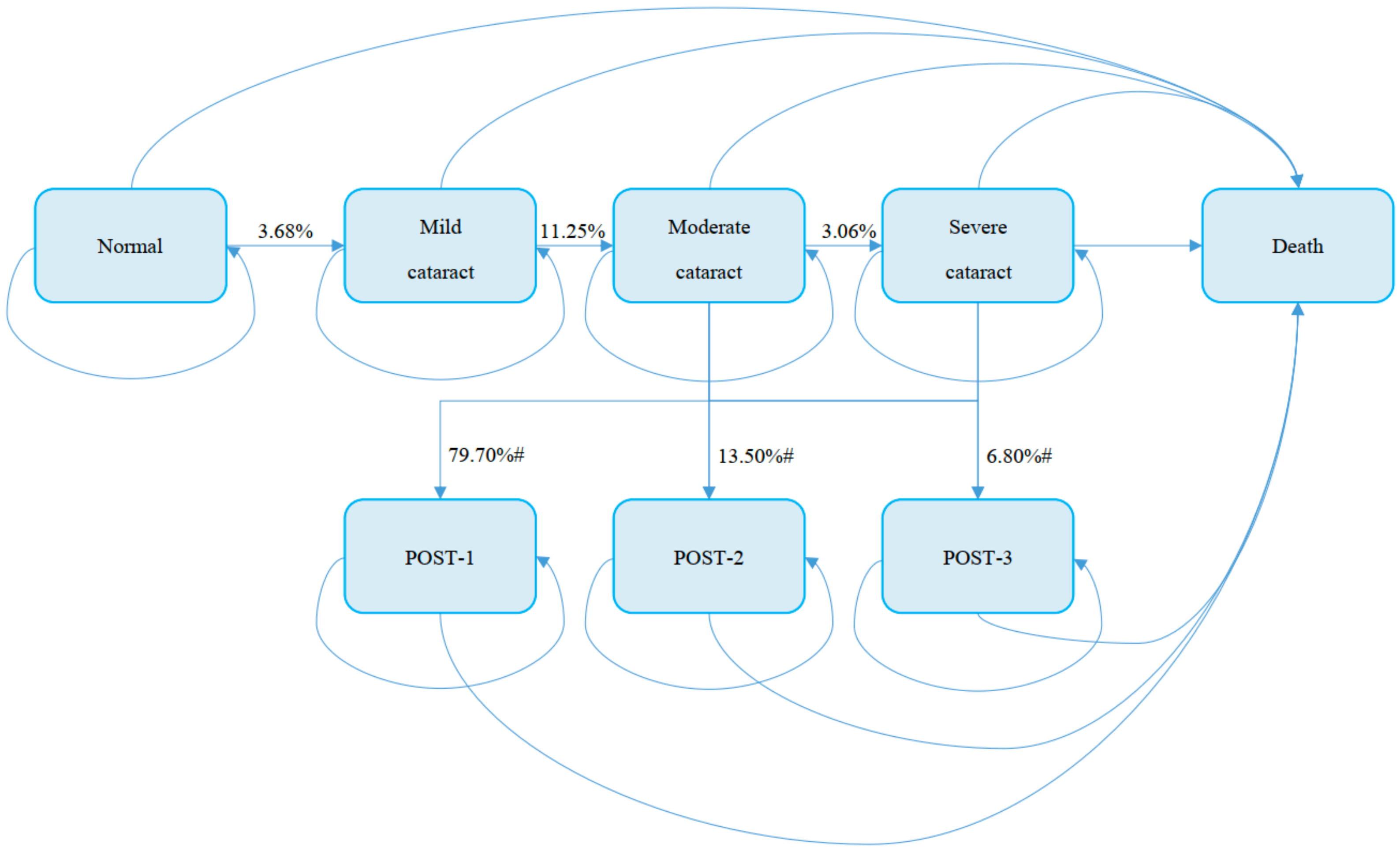

Supplementary Fig.3. Markov models for the natural progression and postoperative distributions of cataracts.

# represents the distributions of postoperative patients in urban setting. The corresponding proportions of POST-1, POST-2, and POST-3 groups in rural setting are 68.6%, 25.32%, and 6.08%, respectively.

Mild cataracts: patients' BCVA before surgery >0•3. Moderate cataracts: patients' BCVA before surgery was within 0•1-0•3. Severe cataracts: patients' BCVA before surgery <0•1. POST-1: patients' postoperative BCVA > 0•3. POST-2: patients' postoperative BCVA was within 0•1-0•3. POST-3: patients' postoperative BCVA < 0•1. BCVA = best corrected visual acuity.

Supplementary References

1. Du, Y.-F. et al. Prevalence of cataract and cataract surgery in urban and rural Chinese populations over 50 years old: a systematic review and Meta-analysis. Int. J. Ophthalmol. 15, 141–149 (2022).
2. Huang, S. Prevalence and Causes of Visual Impairment in Chinese Adults in Urban Southern China: The Liwan Eye Study. Arch. Ophthalmol. 127, 1362 (2009).
3. Tan, X. et al. Impact of cataract screening integrated into establishment of resident health record on surgical output in a rural area of south China. Ann. Transl. Med. 8, 1222–1222 (2020).
4. Huang, W. et al. Five-year incidence and postoperative visual outcome of cataract surgery in urban southern China: the Liwan Eye Study. Invest. Ophthalmol. Vis. Sci. 53, 7936–7942 (2012).
5. Ren, X. et al. Use of cataract surgery in urban Beijing: a post screening follow-up of the elderly with visual impairment due to age-related cataract. Chin. Med. Sci. J. Chung-Kuo Hsueh Ko Hsueh Tsa Chih 30, 1–6 (2015).
6. Li, R. et al. Cost-effectiveness and cost-utility of traditional and telemedicine combined population-based age-related macular degeneration and diabetic retinopathy screening in rural and urban China. Lancet Reg. Health - West. Pac. 23, 100435 (2022).
7. Mathenge, W. et al. Impact of Artificial Intelligence Assessment of Diabetic Retinopathy on Referral Service Uptake in a Low-Resource Setting. Ophthalmology 2, 100168 (2022).
8. Liu, H. et al. Economic evaluation of combined population-based screening for multiple blindness-causing eye diseases in China: a cost-effectiveness analysis. Lancet Glob. Health S2214109X2200554X (2023) doi:10.1016/S2214-109X(22)00554-X.
9. Cyberspace Administration of China. Digital China Development Report (2020). https://www.gov.cn/xinwen/2021-07/03/content_5622668.htm (2021).
10. Wang, D. et al. Use of Eye Care Services among Diabetic Patients in Urban and Rural China. Ophthalmology 117, 1755–1762 (2010).
11. Tang, J. et al. Cost-effectiveness and cost-utility of population-based glaucoma screening in China: a decision-analytic Markov model. Lancet Glob. Health 7, e968–e978 (2019).
12. Eye Care Comparative Effectiveness Research Team (ECCERT) et al. Cost-utility analysis of cataract surgery in Japan: a probabilistic Markov modeling study. Jpn. J. Ophthalmol. 57, 391–401 (2013).
13. National Institute for Health and Care Excellence. Guide to the Methods of Technology Appraisal 2013. (National Institute for Health and Care Excellence (NICE), 2013).
14. Wu, X. et al. Universal artificial intelligence platform for collaborative management of cataracts. Br. J. Ophthalmol. 103, 1553–1560 (2019).
15. China Population Census Yearbook 2020. http://www.stats.gov.cn/tjsj/pcsj/rkpc/7rp/zk/indexch.htm.
16. Zhu, Z., Wang, L., Scheetz, J. & He, M. Age-related cataract and 10-year mortality: the Liwan Eye Study. Acta Ophthalmol. (Copenh.) 98, e328–e332 (2020).
17. Wang, Z., Congdon, N. & Ma, X. Longitudinal associations between self-reported vision impairment and all-cause mortality: a nationally representative cohort study among older Chinese adults. Br. J. Ophthalmol. bjophthalmol-2022-321577 (2022) doi:10.1136/bjo-2022-321577.
