# Supplementary material for: Cost-effectiveness and cost-utility of a digital technology-driven hierarchical healthcare screening pattern in China
Source: Nat Commun. 2024 Apr 30;15:3650. doi: 10.1038/s41467-024-47211-w (PMC11061155; doi:10.1038/s41467-024-47211-w)
Supplement: Supplementary file 2 — Reporting Summary [file 41467_2024_47211_MOESM2_ESM.pdf]

nature portfolio

Corresponding author(s): Yizhi Liu, Rong Pan and Haotian Lin (Last corresponding author)

Last updated by author(s): Nov 30, 2023

Reporting Summary

Nature Portfolio wishes to improve the reproducibility of the work that we publish. This form provides structure for consistency and transparency in reporting. For further information on Nature Portfolio policies, see our Editorial Policies and the Editorial Policy Checklist.

Statistics

For all statistical analyses, confirm that the following items are present in the figure legend, table legend, main text, or Methods section.

n/a	Confirmed	
☐	☒	The exact sample size (*n*) for each experimental group/condition, given as a discrete number and unit of measurement
☐	☒	A statement on whether measurements were taken from distinct samples or whether the same sample was measured repeatedly
☒	☐	The statistical test(s) used AND whether they are one- or two-sided<br>*Only common tests should be described solely by name; describe more complex techniques in the Methods section.*
☒	☐	A description of all covariates tested
☒	☐	A description of any assumptions or corrections, such as tests of normality and adjustment for multiple comparisons
☐	☒	A full description of the statistical parameters including central tendency (e.g. means) or other basic estimates (e.g. regression coefficient) AND variation (e.g. standard deviation) or associated estimates of uncertainty (e.g. confidence intervals)
☒	☐	For null hypothesis testing, the test statistic (e.g. *F*, *t*, *r*) with confidence intervals, effect sizes, degrees of freedom and *P* value noted<br>*Give P values as exact values whenever suitable.*
☐	☒	For Bayesian analysis, information on the choice of priors and Markov chain Monte Carlo settings
☒	☐	For hierarchical and complex designs, identification of the appropriate level for tests and full reporting of outcomes
☒	☐	Estimates of effect sizes (e.g. Cohen's *d*, Pearson's *r*), indicating how they were calculated

Our web collection on statistics for biologists contains articles on many of the points above.

Software and code

Policy information about availability of computer code

Data collection	No sofeware was used for data collection.
Data analysis	All statistical analyses were performed using TreeAge Pro 2022 (Health Version, Williamstown, MA, USA) and the plots were created using Python (version 3.8.13).

For manuscripts utilizing custom algorithms or software that are central to the research but not yet described in published literature, software must be made available to editors and reviewers. We strongly encourage code deposition in a community repository (e.g. GitHub). See the Nature Portfolio guidelines for submitting code & software for further information.

Data

Policy information about availability of data

All manuscripts must include a data availability statement. This statement should provide the following information, where applicable:
- Accession codes, unique identifiers, or web links for publicly available datasets
- A description of any restrictions on data availability
- For clinical datasets or third party data, please ensure that the statement adheres to our policy

All data used to construct the model are publicly available and referenced. All parameters and their values can be found in manuscript and supplementary information. Data used to generate the main and supplementary figures are provided in the source data file.

Research involving human participants, their data, or biological material

Policy information about studies with human participants or human data. See also policy information about sex, gender (identity/presentation), and sexual orientation and race, ethnicity and racism.

Reporting on sex and gender	*Use the terms sex (biological attribute) and gender (shaped by social and cultural circumstances) carefully in order to avoid confusing both terms. Indicate if findings apply to only one sex or gender; describe whether sex and gender were considered in study design; whether sex and/or gender was determined based on self-reporting or assigned and methods used. Provide in the source data disaggregated sex and gender data, where this information has been collected, and if consent has been obtained for sharing of individual-level data; provide overall numbers in this Reporting Summary. Please state if this information has not been collected. Report sex- and gender-based analyses where performed, justify reasons for lack of sex- and gender-based analysis.*
Reporting on race, ethnicity, or other socially relevant groupings	*Please specify the socially constructed or socially relevant categorization variable(s) used in your manuscript and explain why they were used. Please note that such variables should not be used as proxies for other socially constructed/relevant variables (for example, race or ethnicity should not be used as a proxy for socioeconomic status). Provide clear definitions of the relevant terms used, how they were provided (by the participants/respondents, the researchers, or third parties), and the method(s) used to classify people into the different categories (e.g. self-report, census or administrative data, social media data, etc.) Please provide details about how you controlled for confounding variables in your analyses.*
Population characteristics	*Describe the covariate-relevant population characteristics of the human research participants (e.g. age, genotypic information, past and current diagnosis and treatment categories). If you filled out the behavioural & social sciences study design questions and have nothing to add here, write "See above."*
Recruitment	*Describe how participants were recruited. Outline any potential self-selection bias or other biases that may be present and how these are likely to impact results.*
Ethics oversight	*Identify the organization(s) that approved the study protocol.*

Note that full information on the approval of the study protocol must also be provided in the manuscript.

Field-specific reporting

Please select the one below that is the best fit for your research. If you are not sure, read the appropriate sections before making your selection.

☐ Life sciences ☒ Behavioural & social sciences ☐ Ecological, evolutionary & environmental sciences

For a reference copy of the document with all sections, see nature.com/documents/nr-reporting-summary-flat.pdf

Behavioural & social sciences study design

All studies must disclose on these points even when the disclosure is negative.

Study description	A decision-analysis Markov model was constructed to quantitatively analyze different screening strategies for cataracts.
Research sample	The model was built on a simulated cohort of 100,000 residents in urban and rural China, which is a representative simulated population in economic analysis of eye screening in Chinese seniors (references 1-3). Since urban and rural areas are two representative living environments in China, the residents' income level, health conditions, and quality of life vary from each other. Therefore, it is representative to collect the data of corresponding conditions and construct a simulated cohort for studying the future allocation of ophthalmic medical resources in China.<br>Reference:<br>1. Li, R. et al. Cost-effectiveness and cost-utility of traditional and telemedicine combined population-based age-related macular degeneration and diabetic retinopathy screening in rural and urban China. Lancet Reg. Health - West. Pac. 23, 100435 (2022).<br>2. Liu, H. et al. Economic evaluation of combined population-based screening for multiple blindness-causing eye diseases in China: a cost-effectiveness analysis. Lancet Glob. Health S2214109X2200554X (2023) doi:10.1016/S2214-109X(22)00554-X.<br>3. Tang, J. et al. Cost-effectiveness and cost-utility of population-based glaucoma screening in China: a decision-analytic Markov model. Lancet Glob. Health 7, e968–e978 (2019).
Sampling strategy	The model was built on a simulated cohort of 100,000 residents in urban and rural China from 50 years through a total of 30 1-year Markov cycles, which is the representative and common target population based on previous economic evaluations of eye disease screening in Chinese seniors (references 1-3).<br>Reference:<br>1. Li, R. et al. Cost-effectiveness and cost-utility of traditional and telemedicine combined population-based age-related macular degeneration and diabetic retinopathy screening in rural and urban China. Lancet Reg. Health - West. Pac. 23, 100435 (2022).<br>2. Liu, H. et al. Economic evaluation of combined population-based screening for multiple blindness-causing eye diseases in China: a cost-effectiveness analysis. Lancet Glob. Health S2214109X2200554X (2023) doi:10.1016/S2214-109X(22)00554-X.<br>3. Tang, J. et al. Cost-effectiveness and cost-utility of population-based glaucoma screening in China: a decision-analytic Markov model. Lancet Glob. Health 7, e968–e978 (2019).

Data collection	The data were derived published research through internet and collectors were blind to study hypothesis during data collection.
Timing	July 4,2022 to June 18, 2023
Data exclusions	Data exclusions were not included in the study since the analysis was based on simulated population.
Non-participation	In our simulated Markov model, no real participant was involved in the analysis. However, we set the parameter of participation rate in the model development with reference to previous research to mimic real-world drop-out situations(references 1-3).<br>Reference:<br>1. Li, R. et al. Cost-effectiveness and cost-utility of traditional and telemedicine combined population-based age-related macular degeneration and diabetic retinopathy screening in rural and urban China. Lancet Reg. Health - West. Pac. 23, 100435 (2022).<br>2. Liu, H. et al. Economic evaluation of combined population-based screening for multiple blindness-causing eye diseases in China: a cost-effectiveness analysis. Lancet Glob. Health S2214109X2200554X (2023) doi:10.1016/S2214-109X(22)00554-X.<br>3. Tang, J. et al. Cost-effectiveness and cost-utility of population-based glaucoma screening in China: a decision-analytic Markov model. Lancet Glob. Health 7, e968–e978 (2019).
Randomization	Randomization was not relevant to our study because no experimental group assignment was needed in our study.

Reporting for specific materials, systems and methods

We require information from authors about some types of materials, experimental systems and methods used in many studies. Here, indicate whether each material, system or method listed is relevant to your study. If you are not sure if a list item applies to your research, read the appropriate section before selecting a response.

Materials & experimental systems

n/a	Involved in the study
☒	☐ Antibodies
☒	☐ Eukaryotic cell lines
☒	☐ Palaeontology and archaeology
☒	☐ Animals and other organisms
☒	☐ Clinical data
☒	☐ Dual use research of concern
☒	☐ Plants

Methods

n/a	Involved in the study
☒	☐ ChIP-seq
☒	☐ Flow cytometry
☒	☐ MRI-based neuroimaging

Plants

Seed stocks	*Report on the source of all seed stocks or other plant material used. If applicable, state the seed stock centre and catalogue number. If plant specimens were collected from the field, describe the collection location, date and sampling procedures.*
Novel plant genotypes	*Describe the methods by which all novel plant genotypes were produced. This includes those generated by transgenic approaches, gene editing, chemical/radiation-based mutagenesis and hybridization. For transgenic lines, describe the transformation method, the number of independent lines analyzed and the generation upon which experiments were performed. For gene-edited lines, describe the editor used, the endogenous sequence targeted for editing, the targeting guide RNA sequence (if applicable) and how the editor was applied.*
Authentication	*Describe any authentication procedures for each seed stock used or novel genotype generated. Describe any experiments used to assess the effect of a mutation and, where applicable, how potential secondary effects (e.g. second site T-DNA insertions, mosiacism, off-target gene editing) were examined.*
