# Supplementary material for: Cost-effectiveness and cost-utility of a digital technology-driven hierarchical healthcare screening pattern in China
Source: Nat Commun. 2024 Apr 30;15:3650. doi: 10.1038/s41467-024-47211-w (PMC11061155; doi:10.1038/s41467-024-47211-w)
Supplement: Supplementary file 4 — Description of Additional Supplementary Files [file 41467_2024_47211_MOESM4_ESM.pdf]

Title: Supplementary Data 1

Description: The Supplementary Data 1 provides (1) a demo for model construction and corresponding results in “Markov model example” folder, (2) the pseudocode of the algorithms for probabilistic sensitivity analysis in “analysis algorithm” folder, and (3) a README.txt file for guidance.
